# 652. Ceftazidime-avibactam Alone or as Combination Therapy? Multicenter Retrospective Cohort Analysis of Clinical Outcomes in Patients with Carbapenem-resistant Gram-negative Infection

**DOI:** 10.1093/ofid/ofac492.704

**Published:** 2022-12-15

**Authors:** Ahmed Ullah Mishuk, Jeffrey R Strich, Sarah Warner, Junfeng Sun, Seidu Malik, Alexander Lawandi, Maiko Kondo, Michael J Satlin, Aditya Chandorkar, Emily L Heil, Megan K Morales, Anisha Mathur, Joseph Timpone, Darcy Wooten, Daniel Sweeney, Jonathan Pan, Jillian Raybould, Stephanie Bonne, Roberto Colindres, Helen W Boucher, Sara Buckman, Daisuke Furukawa, Daniel Uslan, Samuel F Hohmann, Sameer S Kadri

**Affiliations:** Critical Care Medicine Department, National Institutes of Health Clinical Center, Bethesda, MD; Critical Care Medicine, National Institutes of Health Clinical Center, Bethesda, Maryland; Critical Care Medicine, National Institutes of Health Clinical Center, Bethesda, Maryland; Critical Care Medicine, National Institutes of Health Clinical Center, Bethesda, Maryland; Critical Care Medicine, National Institutes of Health Clinical Center, Bethesda, Maryland; McGill University Health Centre, Mont Royal, Quebec, Canada; Division of Infectious Diseases, Department of Medicine, Lenox Hill Hospital - Northwell Health, New York, New York; Weill Cornell Medical Center, New York, NY; University of Maryland Medical Center, Baltimore, Maryland; University of Maryland School of Pharmacy, Baltimore, Maryland; University of Maryland Medical Center, Baltimore, Maryland; Medstar Georgetown University Hospital, DC, District of Columbia; Medstar Georgetown University Hospital, DC, District of Columbia; Division of Infectious Diseases, University of San Diego Health System, San Diego, California; Division of Pulmonary Critical Care and Sleep Medicine, University of San Diego Health System, San Diego, California; Division of Infectious Diseases, Virginia Commonwealth University, Richmond, Virginia; Virginia Commonwealth University, Richmond, Virginia; Department of Surgery, University Hospital-Newark, Rutgers, The State University of New Jersey, Newark, New Jersey; Tufts Medical Center, Boston, Massachusetts; Tufts Medical Center, Boston, Massachusetts; Department of Surgery, Washington University, St. Louis, Missouri; Division of Infectious Disease, UCLA Medical Center, LA, California; Division of Infectious Disease, UCLA Medical Center, LA, California; Vizient, Center for Advanced Analytic, Chicago, Illinois; National Institutes of Health Clinical Center, Bethesda, Maryland

## Abstract

**Background:**

Ceftazidime-avibactam (caz-avi), a novel β-lactam/β-lactamase inhibitor, is commonly utilized for carbapenem-resistant gram-negative infections (CR-GNI). However, the benefits vs risks of combining caz-avi with other agents are unclear.

**Methods:**

In this retrospective cohort study, inpatients with CR-GNI treated with caz-avi were identified at 9 U.S. hospitals. The impact of caz-avi monotherapy (MT) or combination therapy (CT; i.e., any concomitant use of gram-negative-active antibiotics) was studied using logistic regression, controlling for baseline patient and hospital factors. The primary outcome was in-hospital mortality or discharge to hospice (death), and secondary outcomes were length of stay (LOS), resolution of infectious signs and symptoms (clinical response), 90-day recurrent infection and future caz-avi–resistant organism. An adjusted odds ratio (aOR) with 95% confidence interval (CI) was used to assess the primary and secondary outcomes.

**Results:**

328/499 (65.7%) patients received caz-avi as targeted therapy for a CR-GNI. Overall patients treated with MT and CT were similar at baseline and had comparable baseline demographics although patients treated with CT were more likely to be in the ICU and receive a concomitant empiric in vitro-concordant antibiotic (table 1). The most common organism was *K*lebsiella spp. (44.6%) followed by *Pseudomonas aeruginosa* (27.7%) (table 2). Concomitant gram-negative agents are shown in table 3. Overall, 92 (28.1%) patients died and CT (vs MT) displayed similar adjusted mortality risk (27.7% vs 28.7%; aOR [95%CI]: 0.67 [0.34-1.33]) and LOS (19 [9, 37] and 20 [9, 42.5] days). CT (vs MT) was associated with greater odds of clinical response (aOR: 2.25 [95%CI:1.15-4.41]). Among survivors, similar rates of 90-day recurrent infection (50/154 (32.5%) were observed in CT vs 18/82 (22.0%) in MT group (p=0.09) and 5 (2.19%) patients had future infection with a caz-avi–resistant pathogen (3 in CT and 2 in MT group).

**Figure:**
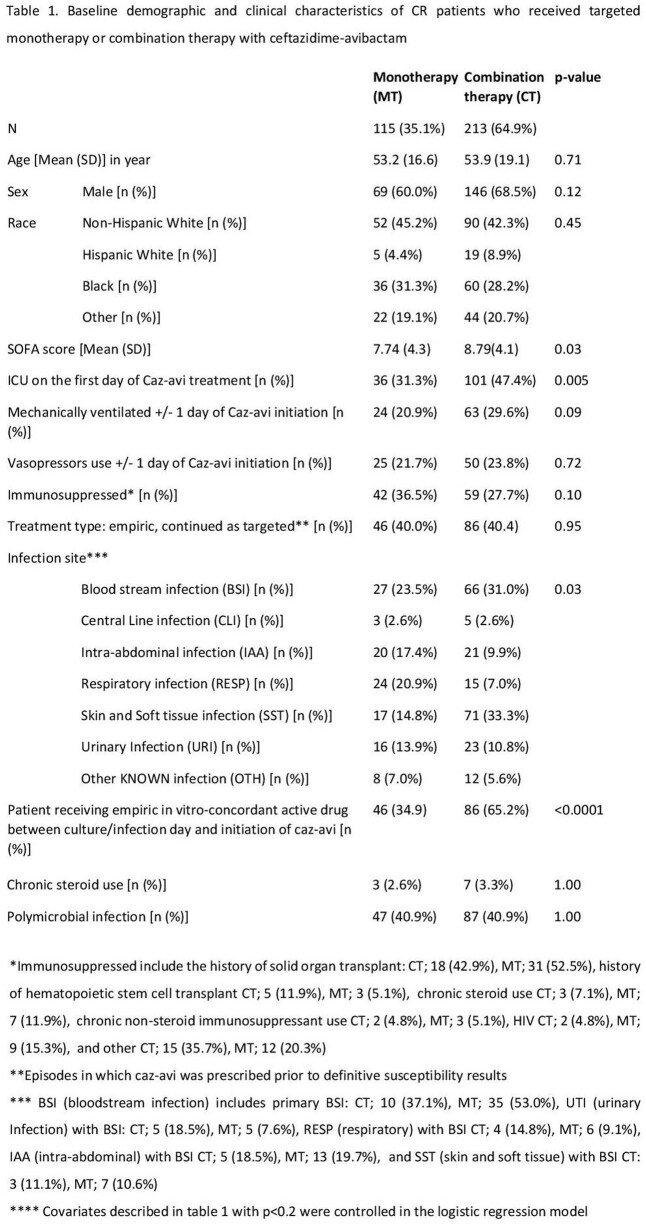

**Figure:**
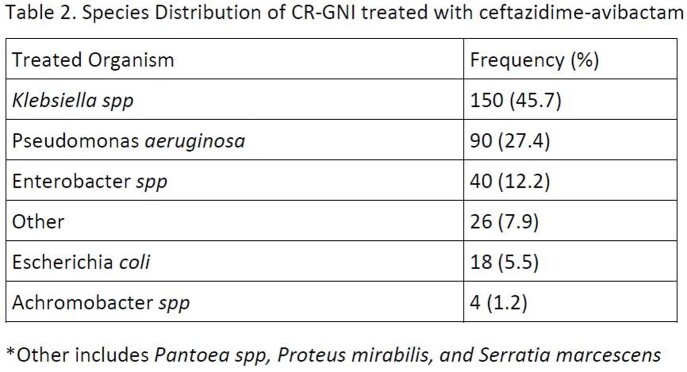

**Figure:**
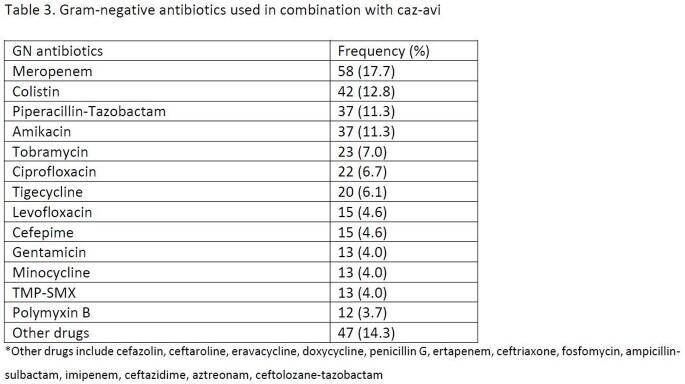

**Conclusion:**

Compared to patients with CR-GNI treated with caz-avi alone, those who received CT including caz-avy had similar survival and LOS but higher clinical response. The role of CT in the era of novel antibiotics warrants additional study.

**Disclosures:**

**Helen W. Boucher, MD**, American Society of Microbiology: Honoraria|Elsevier: Honoraria|Sanford Guide: Honoraria.

